# The Association of Serum Total Peptide YY (PYY) with Obesity and Body Fat Measures in the CODING Study

**DOI:** 10.1371/journal.pone.0095235

**Published:** 2014-04-17

**Authors:** Farrell Cahill, Yunqi Ji, Danny Wadden, Peyvand Amini, Edward Randell, Sudesh Vasdev, Wayne Gulliver, Guang Sun

**Affiliations:** 1 Faculty of Medicine, Memorial University of Newfoundland, St. John’s, Newfoundland, Canada; 2 Discipline of Laboratory Medicine, Memorial University of Newfoundland, St. John’s, Newfoundland, Canada; University of Cordoba, Spain

## Abstract

**Background:**

PYY is an appetite suppressing hormone. Low circulating PYY has been linked to greater BMI. However data is controversial and this association has not been verified in large human populations.

**Objective:**

The purpose of this study was to investigate if fasting serum total PYY is associated with obesity status and/or adiposity at the population level.

**Design:**

A total of 2094 subjects (Male-523, Female-1571) participated in this investigation. Total PYY was measured in fasting serum by enzyme-linked immunosorbent assay. Obesity status (NW-normal-weight, OW-overweight and OB-obese) was determined by the Bray Criteria according to body fat percentage measured by dual-energy x-ray absorptiometry and the WHO criteria according to BMI. One-way ANOVA and multiple regression was used to assess the adiposity-specific association between PYY and the following; weight, BMI, waist-circumference, hip-circumference, waist-hip ratio, percent body fat (%BF), trunk fat (%TF), android fat (%AF) and gynoid fat (%GF).

**Results:**

PYY was not significantly different among NW, OW and OB groups defined by neither %BF nor BMI for both men and women. However among women, fasting PYY was positively associated with adiposity measures. Women with the highest (Top 33%) waist-circumference, %BF and %TF had significantly higher PYY (10.5%, 8.3% and 9.2% respectively) than women with the lowest (Bottom 33%). Age, smoking, medication use and menopause were all positively associated with PYY levels in women but not in men.

**Conclusion:**

To our knowledge this is the largest population based study, with the most comprehensive analysis and measures of confounding factors, to explore the relationship of circulating PYY with obesity. Contrary to initial findings in the literature we discovered that PYY was positively associated with body fat measures (waist-circumference, %BF and %TF) in women. Although the effect size of the positive association of PYY with obesity in women is small, and potentially negligible, it may in fact represent a protective response against significant weight gain.

## Introduction

Peptide YY (PYY), a 36 amino acid appetite suppressing gut hormone, is secreted from the L-cells of the gastrointestinal tract. Low levels of PYY have been reported to be associated with higher BMI and obesity [1]. Circulating PYY increases satiety and subsequently decreases food intake via gut-brain communication [1], [2], [3], inhibits gastrointestinal motility [4] and pancreatic hormone secretion [5], [6]. In addition, it has been documented that PYY plays an integral part in maintaining energy homeostasis [7], [8]. However, the involvement of PYY in the development of human obesity is unclear [9], [10], [11], the relationship between circulating PYY and adiposity is controversial [12], and very little data exists from large human population-based studies.

Appetite, a key factor in energy homeostasis, significantly influences the regulation of body weight [13]. Therefore investigating appetite regulating hormones, such as PYY, may provide valuable insight into the underlying mechanisms responsible for the development of obesity. Initial PYY knockout studies revealed that mice developed significant hyperphagia and that the acute administration of PYY could significantly ameliorate this condition [1], [14], [15]. This group also proposed that endogenous PYY concentration was lower in obese rodents and humans, inversely associated with obesity-related phenotypes [13], and the administration of PYY could effectively reduce food intake and body weight independent of obesity status [1], [13], [14]. Therefore it was hypothesized that a deficiency in circulating PYY could be a significant contributing factor toward the chronic over consumption of food and subsequent weight gain. However, many successive PYY knockout [16], [17] studies have failed to reproduce the strong association of PYY with hyperphegia and diet-induced obesity. A considerable number of studies have also been unable to replicate the original results in rodents or obese humans [11], [18], [19] along with any significant association between circulating PYY and adiposity [6], [20], [21].

Considering that very few cross-sectional studies with large sample sizes have been performed considering confounding variables and that the current results in the literature are controversial; there is a necessity to explore the association of circulating PYY with obesity at the population level. Therefore the purpose of our study was to investigate the association of circulating PYY concentration with obesity status and body composition, measured by dual-energy x-ray absorptiometry, in a large population adjusting for major confounding factors. The detailed objectives were to: 1) test if PYY is associated with obesity status by comparing fasting serum PYY between normal-weight (NW), overweight (OW) and obese (OB) men and women; 2) examine the association of PYY with body composition among normal-weight (NW), overweight (OW) and obese (OB) men and women; and 3) determine the influence of age, sex, smoking, medication use and menopausal status on circulating PYY.

## Methods

### Ethics Statement

This study was approved by The Health Research Ethics Authority (HREA) for the Faculty of Medicine, Memorial University of Newfoundland and Labrador, St John’s, Canada. All subjects provided written informed consent.

### Subjects

A total of 2094 subjects from the Complex Diseases in the Newfoundland population: Environment and Genetics (CODING) study (Male 523, Female 1571) was used for this investigation [22], [23], [24], [25], [26]. All participants of this current study were from the Canadian province of Newfoundland and Labrador. Eligibility of participants for the CODING study was based upon the following inclusion criteria: 1) >19 yrs of age; 2) at least a third generation Newfoundlander; and 3) healthy, without any serious metabolic, cardiovascular, or endocrine diseases. The primary method of subject recruitment for the CODING study was the use of posters and handouts. This literature was distributed throughout public facilities (offices, and hospitals) in the city of St. John’s, Newfoundland and Labrador. Each individual completed a number of questionnaires to obtain information regarding lifestyle and physical activity. Anthropometric, body composition and biochemical measurements were performed following a 12-h fasting period.

### Anthropometric and Body Composition Measurements

Height (cm) and weight (nearest 0.1 kg) measurements were collected and Body Mass Index (BMI) calculated. BMI was defined as weight divided by height squared (kg/m^2^). Waist circumference (cm) was measured as the horizontal distance around the abdomen at the level of the umbilicus, and hip circumference (cm) was measured as the largest circumference between the waist and thighs. Height, waist and hip measurements were recorded to the nearest 0.1 cm. Percent body fat (%BF), percent trunk fat (%TF), percent android (abdominal) fat (%AF) and percent gynoid (lower abdominal-thigh) fat (%GF) were measured, in a supine position, utilizing dual-energy X-ray absorptiometry (DXA, Lunar Prodigy; GE Medical Systems, Madison, WI). The Lunar Prodigy software system determines automatically the regions for the assessment of %TF, %AF, %GF. %TF region is from the top of the shoulders to the top of the iliac crest, while the %AF region is the top of the second lumbar vertebra to the top of the iliac crest and the %GF region extends down iliac crest twice the height of the android area. The current version of the enCORE software for the DXA data presented within this manuscript cannot differentiate visceral from subcutaneous fat. Therefore the %TF, %AF, and %GF regions represent the summation of both subcutaneous and visceral fat relative to these regions. DXA produces an accurate measurement of adipose tissue within the body with a low margin of error. For this reason, DXA is considered to be one of the most reliable measurements of adiposity and is commonly used as a standard compared to less accurate field methods such as BMI. The enCORE (Ver 12.2, 2008, GE Medical Systems, Madison, WI) software package was used for DXA data acquisition. Quality assurance was performed on the DXA scanner daily and the typical CV during the study period was 1.4%.

### Total PYY Measurement

Fasting blood samples were obtained and serum was stored at −80°C for subsequent analyses. Serum total PYY concentration was determined via an enzyme-linked immunosorbent assay (Millipore Corporation Pharmaceuticals, Billerica, MA, USA). Due to the large number of participants in this current investigation samples were measured in singlet. However the intraassay (range of 4.8% to 5.4%) and interassay (5.1%) variation from our previous study, [20] and the measurements of PYY in the current study, were performed by the same research assistant. The detection limit of the PYY enzyme-linked immunosorbent assay used was 10 pg/mL for a sample size of 20 µL.

### Lifestyle Assessment

All participants completed a self-administered screening questionnaire, which queried covariates including smoking (smoker, non-smoker) and medication (medication user, non-medication user). Female subjects were screened for menopausal status (Pre-menopause, Post-menopause) by questionnaire.

### Data Analysis

Data are presented as means ± standard deviation (SD) unless otherwise stated. Participants with PYY concentrations falling outside the range of ±3 SDs (n = 27) were considered outliers and excluded from analyses. The influence(s) of sex (Men vs. Women) environmental factors (smoking vs non-smoking, medication vs non-mediation user) and menopause (pre-menopause vs post-menopause) on PYY concentration were assessed using independent sample t-tests. Pearson correlation analysis was also performed to assess the association of circulating whole PYY with body composition measurements for the entire cohort along with males and females separately. Obesity status (normal-weight, overweight, and obese) for participants (n = 2094) was determined based on %BF according to the, age and sex specific, criteria recommended by Bray [27]. Obesity status was also grouped based on BMI as normal-weight (NW; 18.5–24.9 kg/m^2^), overweight (OW; 25.0–29.9 kg/m^2^) or obese (OB; ≥30 kg/m^2^) according to criteria of the World Health Organization [28]. To further explore the association of PYY with body composition we stratified subjects into tertiles according to waist circumference, hip circumference, waist-hip ratio, BMI, %BF, %TF, %AF and %GF. Participants were also divided into tertiles according to fasting serum PYY concentrations (pg/ml) to examine differences in adiposity measurements. Subsequently, adjusted and unadjusted multiple regression analyses were used to further explore the associations found between body composition measurements and circulating PYY concentration. The effect of age on the relationship between PYY and adiposity was also thoroughly explored. PYY concentrations were compared among normal-weight, overweight, and obese participants, defined by %BF according to the Bray Criteria, from four different age groups: 1) younger than 30 years of age (<30 yrs); 2) 30years of age and greater but less than 40 years of age (≥30 yrs–<40 yrs); 3) 40 years of age and greater but less than 50 years of age (≥40 yrs–<50 yrs); and 4) 50 years of age and greater (≥50 yrs).

Differences in PYY concentration among obesity statuses (NW, OW, OB), body composition tertiles (low, medium, high), were analyzed by one-way analysis of covariance (One-way ANCOVA) controlling for age, sex, smoking, medication use, and menopause. Multiple regression analysis also included age, sex, smoking, medication use, and menopausal status as potential covariates. R statistical software package version 2.15.2 (R development core team) was used for all analyses. Statistical analyses were two-sided and a P value<0.05 was considered to be statistically significant.

## Results

### PYY Concentration and Body Composition Measurements among Male and Female Participants

Body composition characteristics and PYY concentration from both male and female subjects are shown in **Table 1**. Independent t-test analysis revealed that PYY, weight, height, BMI, waist circumference and waist-hip ratio were significantly greater for men than women. Women were significantly greater than men for age, hip circumference, %BF, %TF, %AF, and %GF. Sex differences for body composition measurements among Newfoundland and Labradoreans have been previously described by us [24], [25], [26]. Fasting serum total PYY concentrations were found to be approximately 9%, or 11 pg/ml, higher in men than in women (**Table 1**).

**Table 1:** Body composition characteristics and PYY concentration^1,4,5^.

	Entire Cohort			Male			Female			
	(n = 2094)			(n = 523)			(n = 1571)			
	Mean		SD	Mean		SD	Mean		SD	P
Age (y)^3^	42.77	±	12.8	40.00	±	14.2	43.70	±	12.2	**<0.001**
Weight (kg)^2^	73.49	±	15.3	85.16	±	14.2	69.60	±	13.5	**<0.001**
Height (cm)^2^	165.76	±	8.5	176.12	±	6.4	162.29	±	6.0	**<0.001**
BMI (kg/m^2^)^2^	26.68	±	4.9	27.50	±	4.4	26.40	±	5.0	**<0.001**
Waist (cm)^2^	91.53	±	14.2	96.70	±	13.5	89.80	±	14.0	**<0.001**
Hip (cm)^3^	100.72	±	11.5	99.50	±	10.0	101.10	±	11.9	**<0.003**
Waist-Hip Ratio^2^	0.91	±	0.1	0.97	±	0.1	0.89	±	0.1	**<0.001**
Body fat (%)^3^	34.79	±	9.0	25.40	±	8.0	37.90	±	7.0	**<0.001**
Trunk fat (%)^3^	37.00	±	9.2	30.30	±	8.8	39.20	±	8.1	**<0.001**
Android fat (%)^3^	42.31	±	10.7	36.54	±	10.9	44.21	±	9.9	**<0.001**
Gynoid fat (%)^3^	41.03	±	9.6	29.00	±	7.8	45.03	±	6.1	**<0.001**
Peptide YY (pg/ml)^2^^,^ 4	113.96	±	75.2	122.21	±	77.9	111.21	±	74.1	**<0.004**

1 All values are means ± SDs. Gender differences were analyzed by a one-way ANCOVA.

2 Variable significantly greater in men than women.

3 Variable significantly greater in women than men.

4 PYY Minimum and Maximum (pg/ml) – Entire Cohort (3.7–368.5); Male (7.26–364.7); Female (3.67–368.5).

5 Significance level for one-way ANCOVA (controlling for age) was set to P≤0.05.

### Association of Lifestyle/Environmental Factors with PYY Concentration

The influence of lifestyle and environmental factors such as smoking, medication use, and menopause on circulating PYY concentration were explored. None of these factors had an influence on PYY concentration for men. Female smokers had a 9.7% higher fasting PYY concentration than female non-smokers (120.50±77.6 pg/ml vs 109.87±73.5 pg/ml, p<0.04). PYY concentration was significantly higher for female medication users (114.51±75.5 pg/ml) over non-medications users (106.71±72.4 pg/ml) (p = 0.04). With regards to menopausal status, PYY concentration was 10.3% greater for post-menopausal women over pre-menopausal women (117.78±78.3 pg/ml vs 106.70±71.5 pg/ml, p<0.006). Age, weight, BMI, waist circumference, hip circumference, %BF, %TF, %AF and %GF were all significantly greater for post-menopausal women compared to pre-menopausal women (p<0.004). Lastly, to investigate the potential compound effect of smoking, medication use, and menopausal status on fasting PYY concentration for women we compared post-menopause smoking medication users (n = 53) with pre-menopause non-smoking non-medication users (n = 376). The post-menopause smoking medication users group (139.40±93.7 pg/ml) had a 38.6% higher fasting PYY concentration than pre-menopause non-smoking non-medication users (100.60±67.7)(p<0.005) independent of age, BMI and %BF.

### Pearson Correlation Analysis of Body Composition Measurements with Circulating PYY

Pearson correlation analysis of weight, height, BMI, waist circumference, Hip circumference, waist-to-hip ratio, %BF, %TF, %AF, %GF with circulating PYY are shown in **Table 2**. Although no association was found between any of the body composition measurements with PYY (pg/ml) in men, age (y), weight (kg), height (cm), waist circumference (cm) and the waist-to-hip ratio were significantly positively associated with circulating PYY (pg/ml) in the entire cohort. Additionally, age, waist circumference, hip circumference, %BF, and %TF were significantly positively associated with circulating PYY (pg/ml) in women.

**Table 2:** Pearson correlation of body composition measurements with circulating PYY^1,2^.

	Entire Cohort		Male		Female	
	(n = 2094)		(n = 523)		(n = 1571)	
	r	p	r	p	r	p
Age (y)	0.05	0.031	0.03	NS	0.07	0.009
Weight (kg)	0.05	0.027	−0.03	NS	0.04	NS
Height (cm)	0.05	0.017	0.02	NS	0.01	NS
BMI (kg/m^2^)	0.03	NS	−0.03	NS	0.04	NS
Waist (cm)	0.06	0.003	−0.01	NS	0.07	0.003
Hip (cm)	0.04	NS	−0.04	NS	0.07	0.007
Waist-Hip Ratio	0.06	0.008	0.03	NS	0.03	NS
Body fat (%)	−0.01	NS	−0.02	NS	0.05	0.043
Trunk fat (%)	0.01	NS	−0.01	NS	0.06	0.025
Android fat (%)	0.01	NS	0.00	NS	0.05	0.056
Gynoid fat (%)	−0.05	NS	−0.03	NS	0.01	NS

1 Pearson correlation of body composition measurements with PYY (pg/ml).

2 Statistical significance was set to p<0.05.

### PYY Concentration and Body Composition among Normal-weight, Overweight and Obese for Male and Females Participants

**Figure 1:**
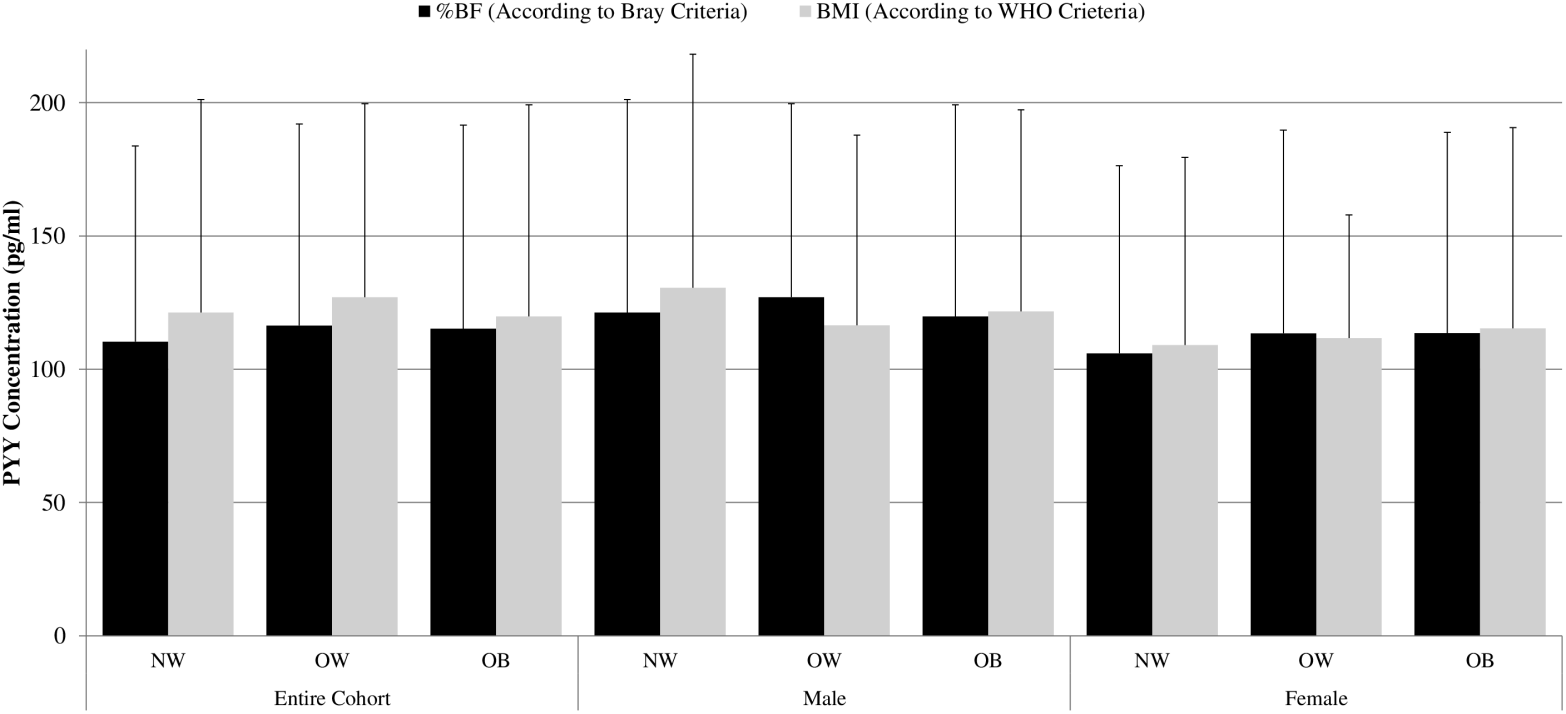
Fasting serum PYY among normal-weight, overweight and obese males and females. Fasting PYY concentration was not significantly different between normal-weight (NW), overweight (OW) or obese (OB) subjects defined either by body mass index (BMI) according to the WHO criteria [28] or percent body fat (%BF) measured by dual-energy x-ray absorptiometry (DXA) according to the Bray criteria [27]. Fasting PYY concentration was also not significantly different among adiposity groups among men and women separately.

Body composition characteristics and PYY for NW, OW and OB men and women are shown in **Table 3**. Fasting total PYY concentration was not significantly different among NW, OW, and OB subjects within the entire cohort or for either sex (**Table 3**). Weight, BMI, waist circumference, hip circumference, waist-hip ratio, %BF, %TF, %AF and %GF all significantly increased with the concomitant increase in adiposity status within the entire cohort and for both sexes. There was also no significant difference in circulating PYY when these adiposity groups were classified by BMI according to the WHO criteria (**Figure 1**).

**Table 3:** Body composition measurements and PYY concentration among NW, OW and OB men and women^1,3^.

	Entire Cohort			Male			Female		
	Normal	Overweight	Obese	Normal	Overweight	Obese	Normal	Overweight	Obese
	(n = 687)	(n = 628)	(n = 779)	(n = 188)	(n = 132)	(n = 203)	(n = 499)	(n = 496)	(n = 576)
Age (y)	40.0±13.8	44.5±12.1	43.8±12.1	37.2±14.9	43.4±12.9	40.4±13.6	41.1±13.2	44.8±11.9	45.0±11.3
Weight (kg)	64.2±10.3	70.6±11.1	84.0±15.6	76.1±8.7	84.3±9.1	94.3±15.3	59.7±6.5	66.9±8.3	80.4±14.0
Height (cm)	166.7±8.6	164.9±7.9	165.6±8.8	176.7±6.6	175.6±5.5	176.9±6.9	164.0±5.9	162.1±5.7	162.0±6.3
BMI (kg/m^2^)	23.0±2.5	25.9±3.0	30.6±4.8	24.4±2.6	27.4±2.7	30.5±4.5	22.5±2.2	25.5±2.9	30.6±4.9
Waist (cm)	81.5±8.7	89.9±10.2	101.7±14.1	87.1±7.9	95.6±7.5	106.3±13.9	79.4±8.1	88.4±10.3	100.0±13.8
Hip (cm)	92.1±7.0	99.4±7.4	109.3±11.2	93.0±7.0	98.5±7.1	106.3±9.6	91.8±7.1	99.6±7.5	110.4±11.5
Waist-Hip Ratio	0.9±0.08	0.9±0.08	0.9±0.09	0.9±0.06	1.0±0.04	1.0±0.09	0.9±0.08	0.9±0.07	0.9±0.07
Body fat (%)	26.5±6.7	35.1±5.5	41.9±6.4	17.4±4.0	25.3±2.1	32.9±3.8	29.9±3.5	37.7±2.1	45.1±3.5
Trunk fat (%)	27.6±6.4	37.7±4.8	44.8±5.7	21.0±5.6	31.1±3.6	38.4±4.0	30.1±4.7	39.4±3.4	47.0±4.3
Android fat (%)	31.7±8.5	43.2±6.1	50.9±6.2	26.0±8.7	37.8±5.8	45.5±5.3	33.9±7.3	44.6±5.3	52.8±5.4
Gynoid fat (%)	34.7±9.0	41.2±7.7	46.5±7.8	22.4±6.4	28.3±4.5	35.6±4.7	39.4±4.2	44.7±3.7	50.3±4.3
Peptide YY (pg/ml)^2^	110.3±73.4	116.4±75.6	115.2±76.4	121.3±79.9	127.0±72.6	119.8±79.4	106.2±70.4	113.5±76.2	113.6±75.3

1 All values are means ± SDs. Subjects were classified on the basis of percentage body fat as normal-weight (NW), overweight (OW), and obese (OB) according to criteria recommended by Bray [27].

2 PYY Minimum and Maximum (pg/ml) – Entire cohort (NW 3.7–359.5, OW 3.7–364.1, OB 4.5–368.5); Male (NW 18.2–358.0, OW 3.7–364.14, 7.3–364.7); Female (NW 3.67–359.5, OW 3.7–364.1, OB 4.5–368.5).

3 Significance level for one-way ANCOVA (controlling for age, gender, smoking, medication use, and menopause) was set to p≤0.05.

### PYY Concentration and Body Composition Measurements among Tertiles (Low, Medium, and High) According to Body Composition Measurements and PYY Concentration

To further explore the potential relationship between PYY and body composition, PYY concentrations were compared among subjects stratified into tertiles according to obesity-related phenotypes (waist circumference, hip circumference, BMI, %BF, %TF, %AF and %GF). We found no significant difference in PYY concentration among any of the body composition measurement tertiles, or a difference in any of the body composition values among the PYY tertiles, in men (**Table 4** **&** **5**). However, PYY concentrations were significantly associated with the increase in waist circumference, %BF, and %TF and these same body composition measurements were significantly associated with the concomitant increase of PYY concentration in women (**Table 4** **&** **5**). PYY concentration was also significantly associated with the increase in waist circumference and waist-hip ratio before and after adjustment for the entire cohort. Hip circumference and waist-hip ratio are also positively associated with the increase in circulating PYY before and after adjustment for the entire cohort and women (**Table 4** **&** **5**). Lastly, %AF was significantly associated with the increase in circulating PYY concentration in females (**Table 4** **&** **5**).

**Table 4:** PYY concentrations according to body composition measurements^1,4,5,6^.

	Entire Cohort					Male					Female				
	Low	Medium	High	P	P*	Low	Medium	High	P	P*	Low	Medium	High	P	P*
Waist (cm)^2^^,^ 3	109.16±73.4	114.65±75.6	118.23±76.4	**0.023**	**0.024**	122.47±82.2	125.34±78.7	118.80±72.5	NS	NS	106.45±70.2	109.77±73.9	117.64±77.9	**0.01**	**0.02**
Hip (cm)	111.71±74.7	115.15±74.4	115.17±76.5	NS	NS	123.79±83.9	123.11±76.1	119.33±73.2	NS	NS	107.60±71.1	110.61±73.3	115.57±77.7	NS	NS
Waist-Hip Ratio	110.46±74.2	110.81±73.8	120.63±77.1	**0.011**	**0.012**	117.54±75.3	121.87±78.8	127.09±79.5	NS	NS	109.29±75.8	108.75±70.4	115.67±76.0	NS	NS
BMI (kg/m^2^)	111.74±75.8	116.85±75.6	113.29±74.1	NS	NS	129.50±85.5	118.13±74.7	118.80±72.6	NS	NS	107.16±71.6	115.64±75.6	110.89±74.9	NS	NS
Body Fat (%)^2^^,^ 3	114.41±73.9	112.90±75.8	114.56±75.9	NS	NS	123.72±81.2	125.61±73.9	117.13±78.5	NS	NS	106.22±70.4	112.48±74.7	115.08±76.9	**0.048**	NS
Trunk Fat (%)^2^^,^ 3	112.84±75.5	113.07±73.2	115.98±76.9	NS	NS	124.64±81.7	122.75±75.1	119.00±77.0	NS	NS	105.92±71.9	112.20±72.0	115.63±78.1	**0.03**	**0.04**
Android fat (%)	112.76±75.8	114.13±74.6	115.00±75.2	NS	NS	122.80±81.1	122.01±74.6	121.65±78.1	NS	NS	106.87±74.7	111.82±72.0	115.06±75.5	NS	NS
Gynoid fat (%)	119.46±76.5	108.85±72.6	113.53±76.1	NS	NS	123.28±80.8	123.55±74.7	119.61±78.3	NS	NS	108.82±71.2	112.10±75.2	112.85±75.9	NS	NS

1 All values are mean ± SDs. Subjects were stratified into tertiles (low, medium and high) based upon body composition measurements.

2 Female - Body Fat % (Low 27.7±2.4, Medium 35.7±2.3, High 44.4±3.6); Trunk Fat % (Low 28.3±3.6, Medium 38.0±2.3, High 46.9±3.6); waist circumference (Low 77.2±5.5 cm, Medium 89.7±3.3 cm, High 107.5±10.1 cm).

3 Male - Body Fat % (Low 86.2±7.6, Medium 95.8±6.9, High 108.3±13.9); Trunk Fat % (Low 85.9±7.6, Medium 96.2±7.8, High 108.39±13.5); waist circumference (Low 83.9±6.1 cm, Medium 95.8±2.3 cm, High 110.9±11.3 cm).

4 P = Differences between sexes and among body composition tertiles were assessed with ANCOVA controlling for age.

5 P* = Differences between sexes and among body composition tertiles were assessed with ANCOVA controlling for age, gender, smoking, medication use, and menopause.

6 Statistical significance level was set to p<0.05.

**Table 5:** Body composition according to PYY concentrations^1,2,3,4,5,6^.

	Entire Cohort					Male					Female				
	Low	Medium	High	P	P*	Low	Medium	High	P	P*	Low	Medium	High	P	P*
	(n = 698)	(n = 700)	(n = 696)			(n = 175)	(n = 174)	(n = 174)			(n = 525)	(n = 522)	(n = 524)		
Waist (cm)	90.06±13.3	92.02±14.6	92.52±14.6	**0.0008**	**0.0006**	96.28±14.3	97.55±13.6	96.36±12.5		NS	88.24±12.6	89.94±14.5	91.23±14.8	**0.0003**	**0.0003**
Hip (cm)	100.02±10.9	100.82±11.8	101.31±11.7	**0.03**	**0.02**	99.53±9.5	99.75±10.6	99.31±9.8	NS	NS	100.16±11.3	101.01±12.2	102.17±12.1	**0.004**	**0.007**
Waist-Hip Ratio	0.90±0.1	0.91±0.1	0.91±0.1	**0.007**	**0.006**	0.97±0.1	0.98±0.1	0.97±0.1	NS	NS	0.88±0.1	0.89±0.1	0.89±0.1	**0.021**	**0.015**
BMI (kg/m^2^)	26.50±4.5	26.64±5.0	26.91±5.0	NS	NS	27.36±4.3	27.71±4.2	27.38±4.6	NS	NS	26.25±4.6	26.27±5.1	26.73±5.1	NS	NS
Body Fat (%)	34.77±9.0	34.86±8.6	34.75±9.2	NS	NS	24.99±8.0	26.25±7.2	24.91±7.4	NS	NS	37.52±7.1	37.79±7.0	38.46±6.9	**0.023**	NS
Trunk Fat (%)	36.69±9.4	37.23±8.9	37.08±9.3	NS	NS	29.61±9.4	31.42±8.5	29.84±8.6	NS	NS	38.65±8.3	39.19±8.0	39.86±8.0	**0.011**	**0.017**
Android fat (%)	41.76±11.1	42.63±10.5	42.53±10.5	NS	NS	35.64±11.8	37.80±10.3	36.17±10.4	NS	NS	43.40±10.1	44.30±9.9	44.99±9.5	**0.007**	**0.017**
Gynoid fat (%)	41.51±9.4	40.95±9.3	40.63±9.9	NS	NS	29.02±8.5	29.63±7.3	28.36±7.5	NS	NS	45.00±6.0	44.88±6.2	45.24±6.1	NS	NS

1 All values are mean ± SDs. Subjects were stratified into tertiles (low, medium and high) based upon PYY Concentration.

2 PYY (pg/ml) - Male (Low 50.3±16.9, Medium 103.8±16.7, High 212.9±62.3); Female (Low 43.2±16.9, Medium 93.9±16.2, High 196.6±60.7).

3 PYY Minimum and Maximum (pg/ml) - Male (Low 7.3–75.3, Medium 75.4–137.6, High 138.1–364.7); Female (Low 3.7–68.6, Medium 68.7–125.5, High 125.6–368.5).

4 P = Differences between among PYY Concentration tertiles were assessed with ANCOVA controlling for age.

5 P* = Differences between among PYY Concentration tertiles were assessed with ANCOVA controlling for age, gender, smoking, and medication use, and menopause.

6 Statistical significance level was set to p<0.05.

### Multiple Regression Analysis of Body Composition Measurements with Circulating PYY

Unadjusted and adjusted linear regression analysis results of %TF, %BF, and waist circumference on circulating PYY are shown in **Table 6**. No association was found between %TF (%) or %BF (%) with PYY (pg/ml) in the entire cohort or among men. Although waist circumference (cm) was not associated with circulating PYY in men, it was positively correlated with the entire cohort before and after the adjustment for age, smoking, medication use and menopausal status. Circulating PYY was positively associated with waist circumference (cm), %TF (%), and %BF (%) in women before adjustment for confounding factors. However after the adjustment for age, smoking, medication use and menopausal status only %TF (%) and waist circumference (cm) remained significantly associated with PYY in women (**Table 6**).

**Table 6:** Multiple Regression for Body Fat (%), Trunk Fat (%), and Waist Circumference (cm) on PYY Concentration^1,3^.

	Unadjusted^2^		Adjusted^2^	
	β	p	β	p
**Body Fat (%)**				
Entire Cohort	−0.1083 (0.183)	NS	−0.2984 (0.195)	NS
Male	−0.1984 (0.451)	NS	−0.1368 (0.476)	NS
Female	0.5417 (0.267)	**0.043**	0.2786 (0.285)	NS
**Trunk Fat (%)**				
Entire Cohort	0.056 (0.179)	NS	−0.1105 (0.194)	NS
Male	−0.1312 (0.386)	NS	−0.1424 (0.421)	NS
Female	0.5170 (0.230)	**0.025**	0.3090 (0.247)	**0.045**
**Waist Circumference**				
Entire Cohort	0.3414 (0.1154)	**0.003**	0.2982 (0.1208)	**0.02**
Male	−0.0720 (0.253)	NS	−0.0943 (0.2692)	NS
Female	0.3930 (0.133)	**0.003**	0.2826 (0.1437)	**0.049**

1 Regression model adjusted for age, gender, smoking, medication use (Menopause was also controlled for in the females).

2 β = Unstandardized Beta (standard error).

3 Statistical significance was set to p<0.05.

### Association of Age with Circulating PYY Concentration

**Figure 2:**
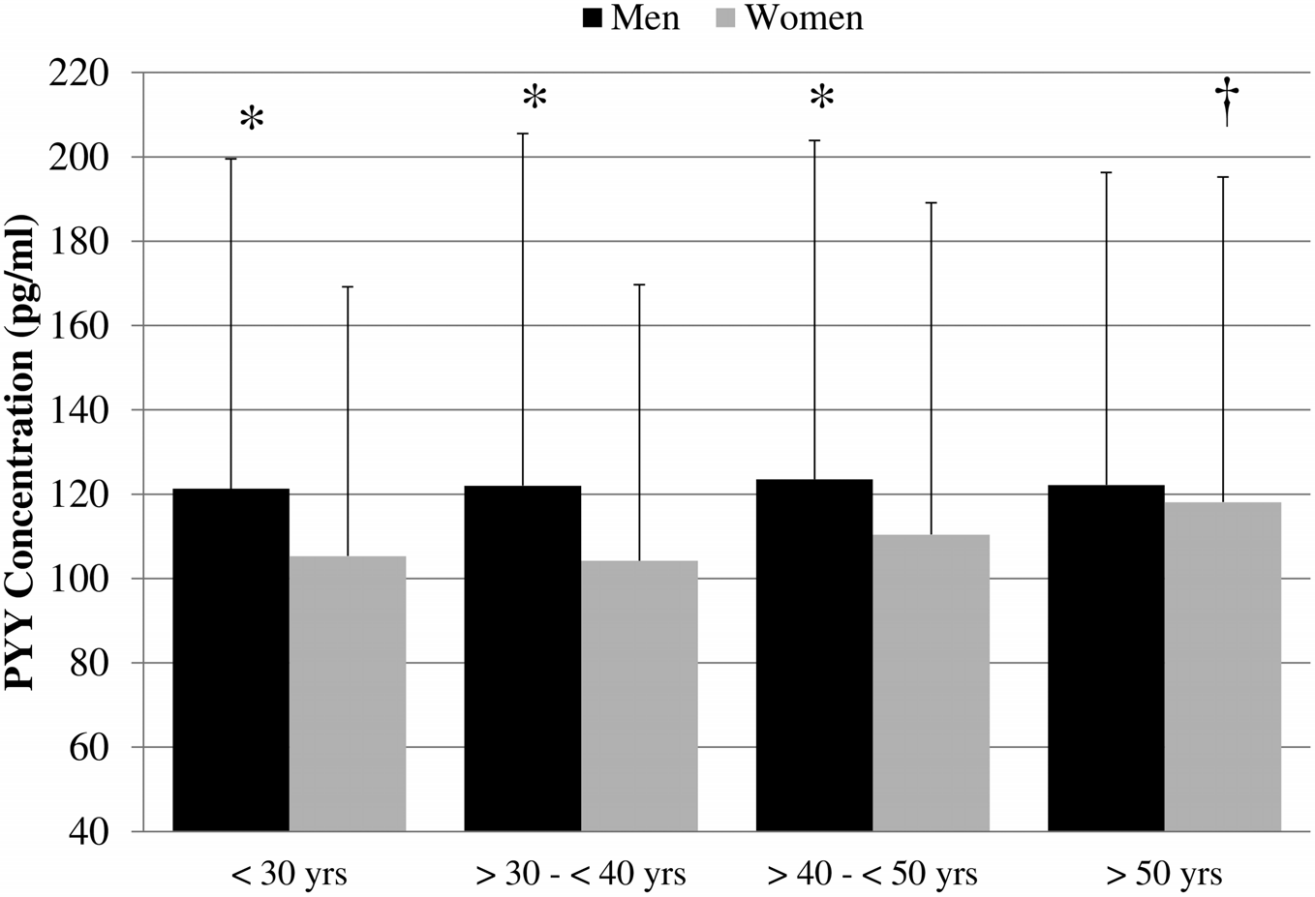
Fasting Serum PYY for men and women in four age groups. Fasting PYY concentration was 15.2%, 17.1% and 11.8% greater among men than women within the <30 yrs, >30–<40 yrs, and >40–<50 yrs groups respectively (*). Additionally, the ≥50 yrs group of women had a 12.2% higher circulating level of PYY than women in the <30 yrs group (†).

Circulating PYY were also analyzed among normal weight, overweight and obese men and women for 4 different age ranges (<30 yrs, ≥30 yrs–<40 yrs, ≥40–<50 yrs, and ≥50 yrs). No sex differences for PYY concentration were found among normal-weight, overweight and obese for any of the 4 age groups (Data no shown) and circulating total PYY concentration was not significantly different among adiposity groups within any of the 4 age ranges (Data no shown). However, PYY concentration was 15.2%, 17.1% and 11.8% greater among men than women within the <30 yrs, >30–<40 yrs, and >40–<50 yrs groups respectively (**Figure 2**.). Additionally, the ≥50 yrs group from women had 12.2% higher circulating PYY than women in the <30 yrs group (118.1±77.1 vs 105.3±63.9) (**Figure 2**).

## Discussion

One major finding for the present study is that serum PYY is not negatively associated with obesity status defined by BMI or %BF adjusting for age, sex, smoking, medication use, and menopause. Contrary to the current literature we have demonstrated for the first time that fasting serum PYY is positively associated with %BF, %TF, and waist circumference in women. Additionally, circulating PYY was greater among men compared to women, and is affected by age, smoking, medication use and menopausal status in women only.

PYY is an anorexigenic peptide, released from the L-cells of the lower intestine, which acts centrally within the hypothalamus to inhibit the orexigenic activity of the neuropeptide Y (NPY) [13], [29]. The initial investigations involving PYY, conducted in rodents, revealed that a diet-induced obesity state decreased PYY concentrations. Suggesting that the circulating concentration of this appetite suppressing gut hormone is associated with the development of obesity [15], [30]. Studies conducted on a small sample of humans showed that endogenous PYY concentrations were lower in obese subjects and inversely correlated with obesity-related phenotypes [13], [15], [31], [32]. However, other rodent [11] and human based [18], [20], [21], [33] studies have failed to reproduce the strong inverse relationship between circulating PYY and adiposity. Additionally, an extensive review in 2005, exploring the anorexigenic effect of PYY, revealed 84% studies produced among 41 independent research groups were unable to reaffirm the claim proposed by the initial investigations [12]. Data from human studies are limited, inconsistent, contain small sample sizes and have utilized less accurate obesity measures (like BMI) in place of more accurate measures of body fat. Moreover, our laboratory and others have shown that BMI cannot accurately distinguish fat mass from fat-free mass and is not an accurate predictor of adiposity [24], [26]. Therefore the inaccurate measurement of adiposity and the misclassifications of obesity status could be the factor attributing to the contradictory reports concerning the association of PYY with obesity. The first important finding from the present study is that circulating PYY was not significantly different among normal-weight, overweight and obese participants and these findings were consistent whether obesity status was defined either by %BF or BMI. Additionally, a recent study by our laboratory was also unable to detect an association of circulating PYY with obesity status (defined either by BMI or %BF) in a cohort consisting of young men [20]. Considering the power of the present study, with over 2000 subjects, our results indicate that circulating PYY concentration is not likely a significant factor affecting obesity status at the population level. However, after extensive analysis we have shown that circulating PYY is positively associated with obesity measures related to body fat in women. PYY concentration was 10.5%, 8.3%, and 9.2% greater among women with the highest waist circumference, %BF and %TF, respectively, compared to women with the lowest aforementioned body fat measures. This finding was also supported by the fact that waist circumference, %BF and %TF were greatest among women with the highest tertile according to PYY concentration and that unadjusted multiple regression analysis revealed a significant positive influence of waist circumference, %BF and %TF. The positive relationship found in our study between PYY and body fat measures are in direct opposition to the negative association theory. However, among the results of a study [34] designed to investigate various metabolites in insulin resistance, we discovered that PYY concentration was greater among obese subjects compared to normal-weight subjects. Although the primary objective of this study was not investigating PYY and obesity status, their results are consistent with our current findings. The increased level of PYY in circulation is likely a protective response of human body to the obese state. Nevertheless, more studies are needed to verify our findings in other populations.

The potential influence(s) of sex, age, smoking, medication use and menopause on fasting serum PYY concentration were also examined. Studies have reported gender differences in circulating PYY levels. One study found that PYY was higher in men compared to women [35], while others found that PYY levels are higher in women [21]. In the present study we discovered that fasting serum PYY was 9% higher in men compared to women. Findings from our current study, along with those of others, suggest that circulating PYY concentration is not significantly affected by age [21], [32], [35]. However, when evaluating the influence of age on PYY among females, a positive association was found. Smoking and medication use did not affect PYY concentration in men, but these factors did significantly increase circulating PYY levels in women. Circulating PYY concentration was 9.7 and 7.3% greater among female smokers and medication users over non-smoker and non-medication users respectively. Currently, there is very little evidence to suggest that smoking and medication use affect circulating PYY. However some data, from animal models, does propose that nicotine [36], [37], [38] and various medications [39], [40], [41] can increase circulating PYY. Specifically, rodent studies have shown that nicotine can significantly increase mRNA expression and the production of PYY protein [36] along with the significant attenuation of neuropeptide Y (NPY) mRNA expression and production of NPY protein [37], [38]. Therefore, it is probable that the inhibition of appetite, due to smoking, could be partially attributed to the stimulation of PYY and the inhibition of NPY in women. Lastly, post-menopausal women had significantly greater PYY than pre-menopausal women. However, since more than 70% of our post-menopausal women are medication users and that menopause is strongly associated with age, the up regulation of PYY could be an age and drug related interference. Therefore we investigated the potential compound effect of smoking, medication use, and menopausal status on fasting PYY concentration for women. The analysis revealed that post-menopause, smoking, medication users had a 38.6% higher fasting PYY concentration than pre-menopause, non-smoking, non-medication users. This compound effect was unchanged after controlling for age, BMI and %BF. Our study provides strong evidence that there is an obvious sex difference regarding fasting PYY concentration and that age, smoking, medication use and menopausal status all significantly influence circulating PYY concentration in women and not men.

The primary limitation of the present study is that we only investigated total PYY rather than PYY_3–36_ alone which has been suggested to be the more significantly active form of PYY. However, total levels of PYY are well correlated with PYY_3–36_ [15] and both PYY_3–36_ and PYY_1–36_ have been found to decrease gastrointestinal motility [6]. Secondly, due to a significant number of subjects in the current study, singlet measurements of PYY were collected from a single serum sample for each subject. However, due to the fact that the intraassay (range of 4.8% to 5.4%) and interassay (5.1%) variation from our previous study [20] were low, and that the measurement of PYY in the current study was performed by the same research assistant, we are confident that our data is accurate. Moreover, the large sample size further strengthens the reliability. Thirdly, although our findings strongly suggest that PYY is not a significant player in determining obesity status, it must be acknowledged that our samples were collected in a fasted state. Population studies, monitoring the dynamic postprandial response of PYY concentration to various standardized diets, should be performed.

In summary, the association of fasting serum PYY concentration with obesity status and body fat measures was investigated among 2094 adults from the Canadian province of Newfoundland and Labrador. To the best of our knowledge this is the largest cross-sectional study to systematically evaluate the relationship between peripheral circulating PYY concentrations with adiposity. Our results indicate that PYY is not significantly associated with obesity status defined by either %BF or BMI. However, circulating PYY is influenced by age, smoking, medication use, and menopausal status in women. Moreover contradictory to previous studies, fasting serum PYY in the present study was significantly associated with waist circumference, %BF, and %TF in women. Although the effect size of the positive association of PYY with adiposity in women is small, and potentially negligible, it may in fact represent a protective response to extreme weight gain.
